# Description of Clinical Characteristics of VAP Patients in MIMIC Database

**DOI:** 10.3389/fphar.2019.00062

**Published:** 2019-02-04

**Authors:** Qingqing Liu, Jin Yang, Jun Zhang, Fanfan Zhao, Xiaojie Feng, Xue Wang, Jun Lyu

**Affiliations:** ^1^Clinical Research Center, The First Affiliated Hospital of Xi’an Jiaotong University, Xi’an, China; ^2^School of Public Health, Xi’an Jiaotong University Health Science Center, Xi’an, China; ^3^ICU, The First Affiliated Hospital of Xi’an Jiaotong University, Xi’an, China

**Keywords:** ventilator-associated pneumonia, VAP, antibiotic, pathogenic bacteria, MIMIC database

## Abstract

**Background:** Ventilator-associated pneumonia (VAP) is a common and serious nosocomial infection of intensive-care units (ICUs). Accurate, timely diagnosis enables early VAP patients to receive appropriate therapies and reduce the occurrence of complication. However, so far clinical datas regarding the epidemiology and mortality of VAP are still limited. Medical Information Mart for Intensive Care (MIMIC) database is a free, open and public resource about ICU research database. MIMIC database is a free, open, public database that collects information on more than 40,000 ICU patients who are predominantly white people. Therefore, the purpose of the present study is to observe and describe the clinical characteristics of VAP patients in ICU from the MIMIC database.

**Method:** A total of 418 patients were enrolled in the study. General information, ventilator use information, microbiology information, antibiotic use information, and some nursing-related information were extracted to describe and analyze the clinical features of VAP patients.

**Results:** The results of the study showed that patients with one or three pathogens were the most. The main pathogens were YEAST (16.71%), STAPH AUREUS COAG+ (11.63%), *Staphylococcus*, COAGULASE NEGATIVE (8.68%), GRAM NEGATIVE ROD (S) (6.14%), and *Pseudomonas aeruginosa* (5.73%). Patients using 4 antibiotics were the most. The top five antibiotics in the largest proportion were synthetic antibacterials (24.66%), peptides (20.13%), cephalosporins (19.60%), penicillins (13.54%), and aminoglycosides (5.27%).

**Conclusion:** This study summarizes the common pathogens of VAP and the antibiotics commonly used in the treatment of VAP by describing the clinical information of 418 patients with VAP in the MIMIC database. In clinical treatment, we should pay attention to aseptic operation, develop appropriate antibacterial measures, closely monitor the pathogens of VAP infection, and use antibiotics in a timely manner to control the occurrence and development of VAP.

## Introduction

Ventilator-associated pneumonia (VAP) is a common and serious nosocomial infection of intensive-care units (ICUs) (Legras et al., 1998; Urli et al., 2002). The incidence of VAP is 15.6% worldwide, among which the highest incidence is 19.4% in Europe, followed by 16.0% in Asia Pacific, 13.8% in Latin America, and 13.5% in the United States (Kollef et al., 2014). The occurrence of VAP not only prolongs ventilatory support, but also prolongs the stays in ICU and hospital, which would increase healthcare cost and result in a poorer prognosis (Kollef et al., 2012; Craven et al., 2013; Mathai et al., 2015).

Antibiotics are important means for the treatment of VAP. As it is previously reported, early VAP is associated with more antibiotic-sensitive microorganisms, while resistant strains are frequently observed with late-onset VAP (Tseng et al., 2012). Because of the emergence of resistant strains, VAP remains a major cause of morbidity and mortality among critically ill patients and incurs excessive medical expenses for institutions. Accurate, timely diagnosis enables early VAP patients to receive appropriate therapies and reduce the occurrence of complication. However, so far clinical datas regarding the epidemiology and mortality of VAP are still limited (Yue et al., 2015).

Medical Information Mart for Intensive Care (MIMIC) database is a free, open and public resource about ICU research database. MIMIC database was published in 2006 by the computational physiology laboratory of the Massachusetts Institute of Technology (MIT), the Beth Israel Dikang Medical Center (BIDMC) and Philips Medical Center. It has attracted more and more researchers from academia and industry to utilize the database for medical research (Dejam et al., 2014; Lee et al., 2015). Therefore, the purpose of the present study is to observe and describe the clinical characteristics of VAP patients in ICU from the MIMIC database.

## Materials and Methods

### Data Sources

Funded by the National Institutes of Health (NIH), the MIMIC database was jointly published by the Computational Physiology Laboratory of MIT, Beth Israel Dikon Medical Center, and Philips Medical, it Collected and sorted out the clinical diagnosis and treatment information on more than 40,000 real patients who are predominantly white people living in the intensive care unit of the Beacon Israel Dikang Medical Center from 2001 to 2012 (Saeed et al., 2011). The database has well solved the current situation of clinical medical workers suffering from the scientific research without a large number of systematic clinical diagnosis and treatment data. The MIMIC database is constantly updated and the latest version is currently MIMIC-III v1.4 (Zhang, 2015; Johnson et al., 2016). This study mainly builds the library and extracts the information data of relevant patients from the MIMIC database through PostgreSQL software (version 9.6).

### Research Method

We extracted the hadm_id of 564 person-times inpatients with VAP diagnosis from the MIMIC database by using of icd9 code (99731: Ventilator associated pneumonia). To ensure the association of cases with VAP, we searched for VAP-related keywords (VAP or “ventilator-associated pneumonia” or “venting-associated pneumonia”) in the noteevents of these cases, the number of cases with keywords was 418 person-times (407 patients, 9 of whom were hospitalized 2 times, and 1 was hospitalized 3 times). The final search was performed on November 2, 2018. These 418-person-time patients are the subject of our research. Taking hadm_id of 418 person-times as index, we extracted the patient’s general information, ventilator use information, microbiology information, antibiotic use information, and some nursing related information from the corresponding tables to describe and analyze the clinical characteristics of VAP patients. The project was approved by the institutional review boards of the MIT and Beth Israel Deaconess Medical Center (BIDMC) and was granted a waiver of informed consent.

### Data Processing

Continuous variables were expressed as mean ± SD, the distribution of data was represented by pie charts, and the specific states of different indicators were displayed in a bar chart.

## Results

Basic situation of MIMIC-III database: The clinical data were derived from the Internal Medicine ICU (MICU), Cardiac Surgery ICU (CSRU), Surgical ICU (SICU), Cardiology ICU (CCU), Trauma Surgery ICU (TSICU), and Neonatal ICU (NICU), a total of 26 tables including patient demographics, laboratory test results, drug use records, medical records, and information on out-of-hospital deaths, physiological waveforms, etc., such as: ADMISSIONS, CHARTEVENTS, D-ITEMS, DIAGNOSES_ICD, etc.

A total of 418 patients were included in the study. Table 1 summarized the basic characteristics of males and females in baseline data. The average age of male patients was 58.22 years and that of female patients was 64.63 years. The proportion of white individuals was the highest among males and females, followed by blacks. In terms of marital status, the proportion of married people was the highest, followed by singles, same situation for both males and females. In terms of insurance, there were a total of five types of insurance among these patients. The proportion of these insurances, in descending order: Medicare, Private, Medicaid, Government, and Self Pay. In terms of oral care, the head of a bed up, parenteral nutrition, and Sputum suction, the proportion of patients who have not taken measures were larger. The average using time for ventilator was 7.59 h of male patients and was 6.67 h of females. Both males and females had a highest proportion of survival.

**Table 1:** Basic characters of study participants.

	Male (*n* = 249)	Female (*n* = 169)
**Age (mean ± SD)**	58.22 ± 17.25	64.63 ± 16.19
**Race (%)**		
Asian	8 (3.2)	6 (3.6)
Black	18 (7.2)	23 (13.6)
White	182 (73.1)	109 (64.5)
Other	10 (4.1)	9 (5.3)
Unknown	31 (12.4)	22 (13)
**Marital status (%)**		
Married	111 (44.6)	62 (36.7)
Divorced	15 (6)	18(10.7)
Separated	3 (1.2)	2 (1.2)
Single	99 (39.8)	43 (25.4)
Widowed	3 (1.2)	32 (18.9)
Unknown	18 (7.2)	12 (7.1)
**Insurance type (%)**		
Medicaid	31 (12.4)	19 (11.2)
Private	98 (39.4)	43 (25.4)
Medicare	109 (43.8)	102 (60.4)
Government	9 (3.6)	4 (2.4)
Self Pay	2 (0.8)	1 (0.6)
**Oral care (%)**		
Yes	77 (30.9)	43 (25.4)
No	172 (69.1)	126 (74.6)
**Sputum suction (%)**		
Yes	13 (5.2)	7 (4.1)
No	236 (94.8)	162 (95.9)
**Parenteral nutrition (%)**		
Yes	20 (8)	13 (7.7)
No	229 (92)	156 (92.3)
**The head of a bed up (%)**		
Yes	8 (3.2)	7 (4.1)
No	241 (96.8)	162 (95.9)
**Total ventilator time (h)**	7.59 (7.37)	6.67 (6.88)
**Ending situation (%)**		
Death	33 (13.3)	36 (21.3)
Survive	216 (86.7)	133 (78.7)


**FIGURE 1:**
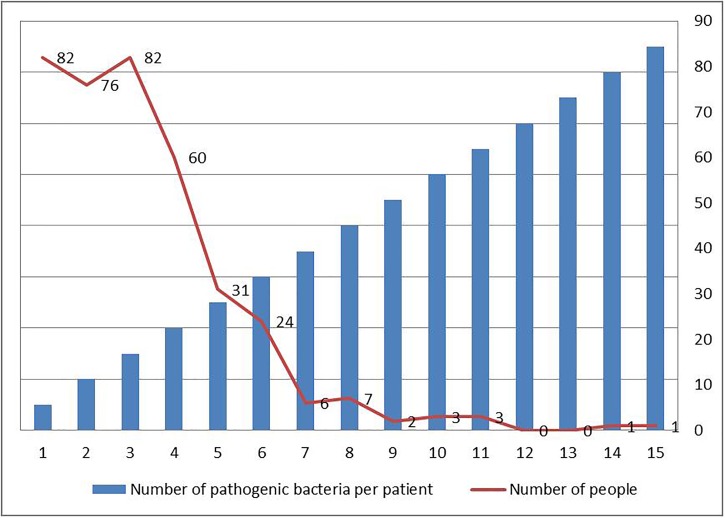
Distribution of number of pathogenic bacteria in each VAP patients in intensive care medical database III (MIMIC-III).

**FIGURE 2:**
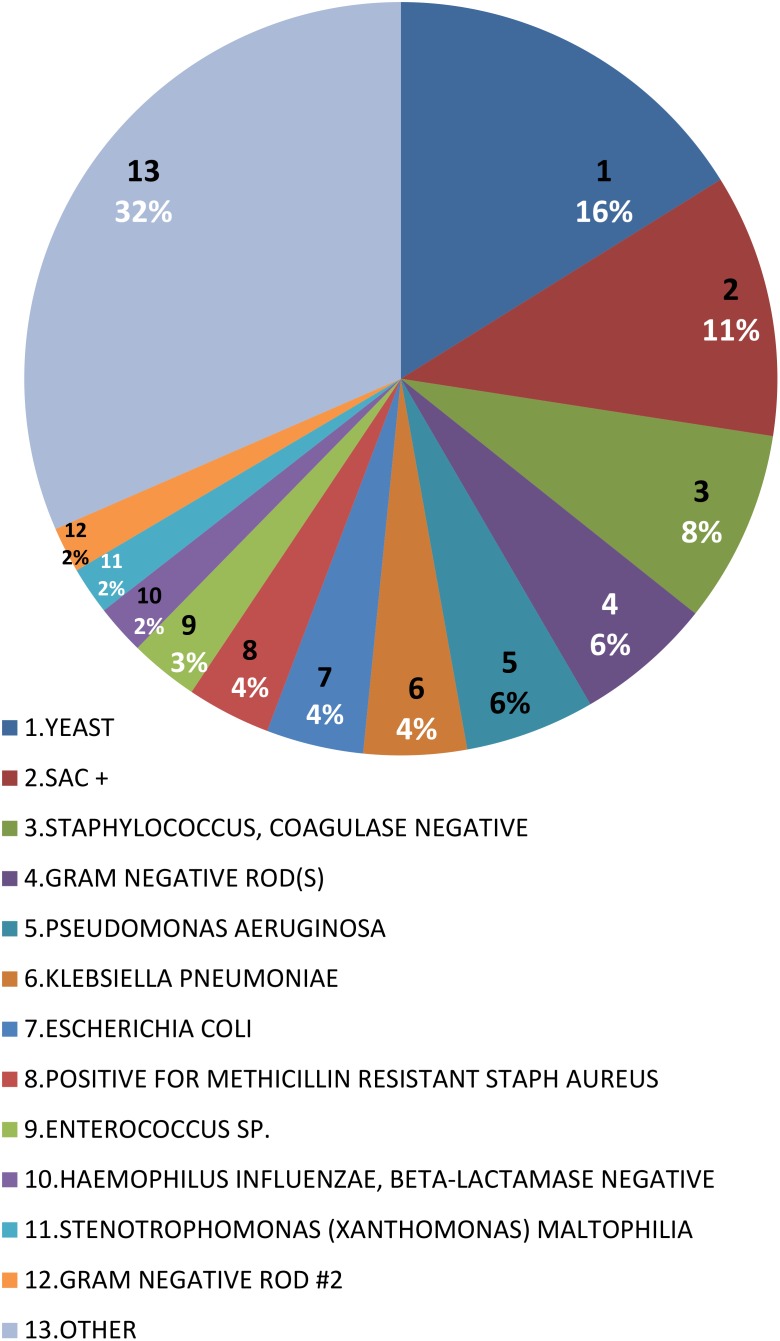
Distribution of bacterial species in VAP patients in intensive care medical database III (MIMIC-III).

Figure 1 summarized the number of pathogenic bacteria carried in each patient. There were 82 patients carried only one pathogen, 76 patients carried two pathogens, and 82 carried three pathogens. One patient carried 15 pathogens, more than any single patient. Figure 2 summarized the proportion of various pathogens in these VAP patients. The top five pathogens with the highest proportion, in descending order: YEAST (16.71%), STAPH AUREUS COAG+ (11.63%), *Staphylococcus*, COAGULASE NEGATIVE (8.68%), GRAM NEGATIVE ROD (S) (6.14%), and *Pseudomonas aeruginosa* (5.73%), the rest were other pathogenic bacteria (52.83%).

**FIGURE 3:**
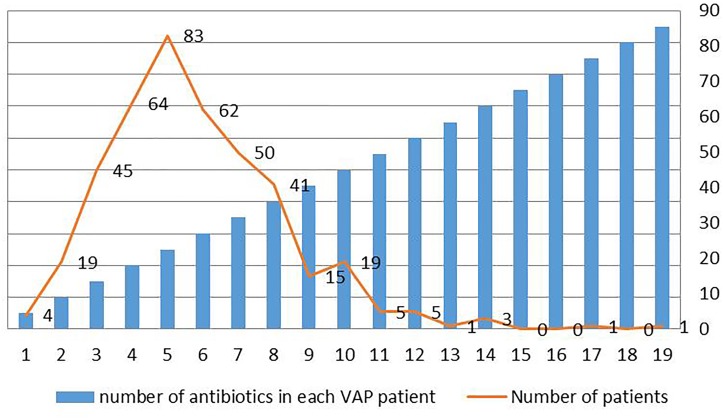
Distribution of number of antibiotics in each VAP patient in intensive care medical database III (MIMIC-III).

Figure 3 summarized the number of antibiotics used in each patient. Four people used only one antibiotic, and one person used 19 antibiotics, which was the patient with the highest number of antibiotics. The largest number of patients used 5 antibiotics, a total of 83 people.

**FIGURE 4:**
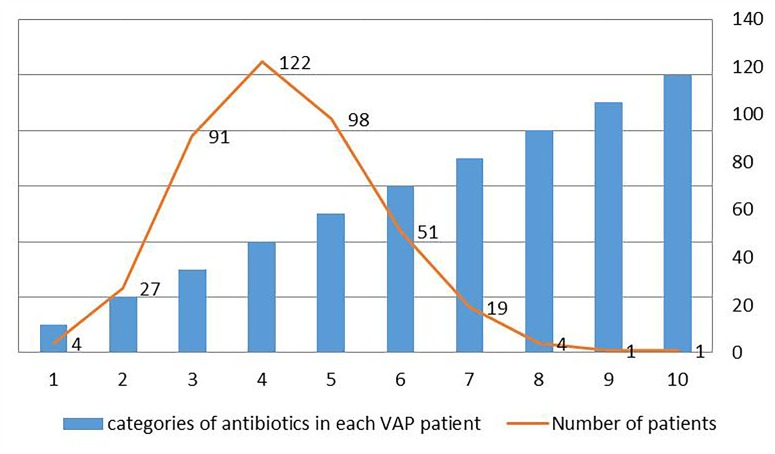
Distribution of categories of antibiotics in each VAP patient in intensive care medical database III (MIMIC-III).

**FIGURE 5:**
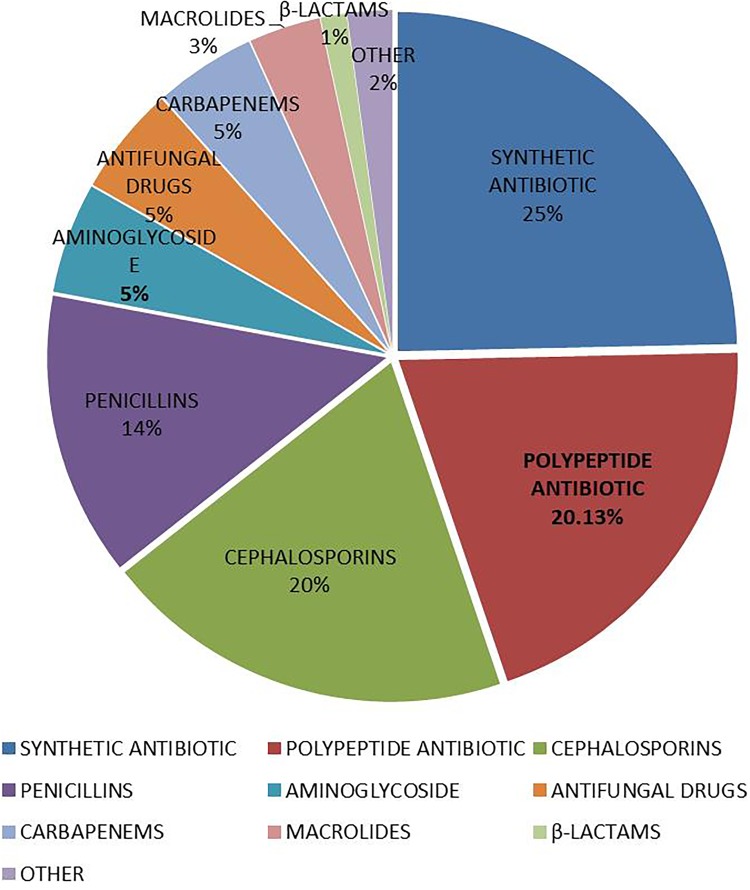
Distribution of categories of antibiotics in VAP patients in intensive care medical database III (MIMIC-III).

Figure 4 summarized the categories of antibiotic used by each patient. Four people used only one categories of antibiotics, and one used 10 categories of antibiotics, which was the patient with the most categories of antibiotics. The largest number of patients used 4 categories of antibiotics, a total of 122 people. Figure 5 summarized the proportion of each categorie of antibiotic in these VAP patients. The top five antibiotics in the largest proportion were, in order, synthetic antibacterials (24.66%), peptides (20.13%), cephalosporins (19.60%), penicillins (13.54%), and aminoglycosides (5.27%), the rest were other antibiotics (16.80%), mainly including antifungal drugs, carbapenems, macrolides, β-lactams, antituberculosis drugs and anti-leprosy drugs, lincomycin, clindamycin, and tetracyclines.

## Discussion

In this study, the basic information and medication status of patients with VAP were extracted and described in the latest version of the multi-parameter intensive care database MIMIC-III. The MIMIC-III database provides relevant data reference for clinicians and engineers who want to solve clinical research problems. This study is mainly to describe the bacterial infection and antibiotic use in patients with VAP. The results of the study showed that patients with one or three pathogens were the most. The main pathogens were YEAST, STAPH AUREUS COAG+, *Staphylococcus*, COAGULASE NEGATIVE, GRAM NEGATIVE ROD (S) and *Pseudomonas aeruginosa*. Patients using 4 antibiotics were the most. The top five antibiotics in the largest proportion were synthetic antibacterials (24.66%), peptides (20.13%), cephalosporins (19.60%), penicillins (13.54%), and aminoglycosides (5.27%).

Mechanical ventilation is an important measure to rescue critically ill patients. VAP is a common complication in mechanical ventilation and a special type of hospital-acquired pneumonia. Patients admitted to the ICU are critically ill and have low immunity. Various invasive procedures create conditions for bacterial infection. In order to prevent the occurrence of stress ulcers, severe patients often use antacids to increase the pH level of gastric juice and create conditions for bacterial growth. Mechanical ventilation destroys the defense barrier of the respiratory tract, making it easy for exogenous bacteria and pathogenic bacteria in the body to enter the bronchial and lung tissues. This makes the lungs susceptible to infection by pathogenic bacteria.

Toxins and invasive enzymes secreted by these bacteria can destroy the body’s normal immune activity by damaging human white blood cells and macrophages, leading to VAP (Kradin and Digumarthy, 2017). The common pathogens of VAP are STAPH AUREUS COAG+, *Staphylococcus*, GRAM NEGATIVE ROD (S), and *Pseudomonas aeruginosa* (Djordjevic et al., 2017), which are consistent with the results of our study.

The occurrence of VAP in ICU patients is responsible for greater morbidity, prolonged ICU stay, and exceeded medical expenses (Vandana Kalwaje and Rello, 2018). On a per case basis, case VAP is associated with additional hospital costs of approximately US$ 40,000 (Warren et al., 2003; Zimlichman et al., 2013). Based on the timing of onset, VAP can be divided into early-onset VAP, occurring within4 to 5 days after intubation and mainly caused by community pathogens with a favorable pattern of antibiotic sensitivity (e.g., *Haemophilus influenzae*, *Streptococcus pneumoniae*, and anaerobes of the oral cavity), and late-onset VAP, often caused by multidrug-resistant (MDR) pathogens (e.g., *Staphylococcus aureus*, *Pseudomonas aeruginosa*, *Acinetobacter baumannii*, and Enterobacteriaceae), which were selected by exposure to broad-spectrum antibiotics (Giard et al., 2008; Shorr et al., 2009).

Antibiotics are the most important means of preventing and treating VAP, so it is important to determine the early diagnosis of VAP and early antibiotic treatment. However, early diagnosis of VAP has become difficult due to the early lack of microbiological analysis and highly specific radiological signs of VAP (Bassetti et al., 2012). Therefore, the adequacy of empirical antibacterial therapy in the early stage is highly predictive of hospital survival (Agrafiotis et al., 2011). For patients with VAP who have a microbiologically determined diagnosis, appropriate broad-spectrum antibiotics must be administered immediately. In our study, a total of 418 hospitalizations of 407 VAP patients in MIMIC database were analyzed. These VAP patients received a total of 13 antibiotic treatments, and the top five antibiotics with the highest frequency were synthetic antibacterials (24.66%), peptides (20.13%), cephalosporins (19.60%), penicillins (13.54%), and aminoglycosides (5.27%), others were followed by antifungal agents (5.19%), carbapenems (4.82%), macrolides (3.42%), beta lactams (1.24%), antituberculosis drugs and anti-leprosy drugs (1.24%), lincomycin and clindamycin (0.49%), and tetracyclines (0.37%). These antibiotics kill bacteria by destroying their cell walls or cell membranes (Běhal, 2002). The results of this study directly reflect the antibiotic use of VAP patients in the MIMIC-III database.

The MIMIC-III database is the first large ICU database that is open to the public free of charge. It has a real large data set with rich types of medical data, providing high-quality data resources for clinical research, mining and building a knowledge base. Effectively assist clinical decision making. This study was subject to some limitations: First, we extract data from MIMIC data, which is a retrospective study with selective bias. Second, this study mainly involved white people, blacks and Asians are very few. Due to ethnic differences, the results of this study need to be further verified in other ethnic groups. Third, we did not conduct influencing factor analysis and predictive analysis for this disease. Influencing factors analysis and predictive analysis can better remind people how to prevent the disease from onset; predict the progression of the disease according to the patient’s condition and basic physiological characteristics, provide patients with better prognosis and rational allocation of medical resources. This will be the main direction of our next research.

## Conclusion

This study summarizes the common pathogens of VAP and the antibiotics commonly used in the treatment of VAP by describing the clinical information of 418 patients with VAP in the MIMIC database. In clinical treatment, we should pay attention to aseptic operation, develop appropriate antibacterial measures, closely monitor the pathogens of VAP infection, and use antibiotics in a timely manner to control the occurrence and development of VAP.

## Author Contributions

All authors listed have made a substantial, direct and intellectual contribution to the work, and approved it for publication.

## Conflict of Interest Statement

The authors declare that the research was conducted in the absence of any commercial or financial relationships that could be construed as a potential conflict of interest.
